# The applicability research of the diagnostic criteria for 6.7.2 angiography headache in the international classification of headache disorders-3rd edition

**DOI:** 10.1186/s10194-021-01373-w

**Published:** 2022-01-31

**Authors:** Chenglong Lu, Leyi Zhang, Jun Wang, Xiangyu Cao, Xin Jia, Xiaohui Ma, Ran Zhang, Lin Wang, Ying Yang, Fanchao Meng, Shengyuan Yu, Ruozhuo Liu

**Affiliations:** 1 Medical School of Chinese PLA, Beijing, 100853 People’s Republic of China; 2 Department of Neurology, the First Medical Centre of Chinese PLA General Hospital, Fuxing Road 28, Haidian District, Beijing, 100853 People’s Republic of China; 3 Department of Vascular and Endovascular Surgery, the First Medical Centre of Chinese PLA General Hospital, Beijing, 100853 People’s Republic of China; 4 Department of Cardiology, the First Medical Centre of Chinese PLA General Hospital, Beijing, 100853 People’s Republic of China

**Keywords:** ICHD-3, Diagnostic criteria, Digital subtraction angiography (DSA), Headache, Angiography, Endovascular procedures, Cerebral angiography headache, Headache due to coronary intervention, Headache due to extremities arterial intervention

## Abstract

**Background:**

Angiography headache (AH) is common but not negligible, and the criteria for AH have been based on only a few studies. The purpose of this study was to investigate the incidence, risk factors and possible mechanism of AH and reappraise the diagnostic criteria for AH in the International Classification of Headache Disorders 3 (ICHD-3).

**Methods:**

Two hundred and seventy-nine patients completed this prospective, non-randomized study, including 107 patients who underwent cerebral angiography, 101 patients who underwent coronary intervention and 71 patients who underwent extremities arterial intervention. Patients were followed up with questionnaires immediately after the procedure and 24 h, 72 h, 1 week and 2 weeks after the procedure.

**Results:**

The incidence of headache was 22.4% (24/107) in cerebral angiography group, 23.8% (24/101) in coronary intervention group, and 16.9% (12/71) in extremities arterial intervention group. Headache still occurred in 12.1% (13/107), 14.9% (15/101) and 11.3% (8/71) of patients 24 h after the procedure in the three groups, respectively. Two types of headache were observed in cerebral angiography group and coronary intervention group, one during and one after the procedure, while only postoperative headache was observed in extremities arterial intervention group. Previous headache history was a risk factor for headache in the three groups (*p* = 0.003 in cerebral angiography group, *p* = 0.006 in coronary intervention group, and *p* = 0.016 in extremities arterial intervention group). In addition, female (*p* = 0.008) was a risk factor for cerebral angiography group. Headache characteristics were described in detail.

**Conclusions:**

The diagnostic criteria for *6.7.2 angiography headache* in ICHD-3 may miss a number of cerebral AH with onset later than 24 h after the procedure. Therefore, it is recommended to revise it according to the literature and further studies. The incidence of headache was high during and after angiography and interventional procedure. It was suggested that the definition of headache due to coronary intervention and headache due to extremities arterial intervention should be added in ICHD.

## Background

Previous studies have reported that the incidence of headache in cerebral angiography ranges from 6.9–55.6% [1–6]. The possible mechanisms for angiography related headache are irritation of the trigeminovascular system by contrast agents or mechanical stimuli such as the catheter itself, activation of platelets or irritation of the vascular endothelium, and selective relaxation and dilation of intracranial vessels [1, 2, 7–10]. ICHD-3 version published in 2018, the diagnostic criteria for AH (Chapter 6.7.2) is showed in Table 1. The criteria defined the onset of cerebral AH to no more than 24 h after cerebral angiography and headache resolved within 72 h after the angiography. In clinical practice we found that headache duration was less than 24 h in many patients with cerebral AH. Nearly half of patients still had headaches after 24 h of cerebral angiography, and about a third of patients have more than one headache after cerebral angiography. These phenomena did not meet the diagnosis criterion of cerebral AH in ICHD-3. In addition, Ramadan proposed the mechanism of headache may be that the catheter or the contrast material activate platelets or damage the vascular endothelium thereby resulting in the release of vasoactive substances such as nitric oxide (NO) or serotonin (5-HT). Should this hypothesis be true, the peripheral or coronary angiography will have the same headache incidence as the cerebral angiography [2].

**Table 1:** International classification of headache disorders-3

Section 6.7.2: Angiography headache	
Description: Headache caused directly by cerebral angiography.	
Diagnostic criteria:	
A. Any new headache fulfilling criterion C	
B. Intra-arterial carotid or vertebral angiography has been performed	
C. Evidence of causation demonstrated by at least two of the following:	
1. headache has developed during or within 24 hours of the angiography	
2. headache has resolved within 72 hours after the angiography	
3. headache has one of the following sets of characteristics^1^:	
a) developing during contrast injection and lasting <1 hour	
b) developing a few hours after the angiography and lasting >24 hours	
c) occurring in a patient with 1. Migraine and having the features of 1.1 Migraine without aura or 1.2 Migraine with aura	
D. Not better accounted for by another ICHD-3 diagnosis.	

^1^There are three recognized (but not separately coded) subforms of 6.7.2 Angiography headache

a) occurring during angiography, and closely related to contrast injection

b) occurring later, but within 24 hours (both these subforms are more common in patients with a history of primary headache, but are distinctly different in character from the primary headache)

c) a migraine attack, occurring in a person who has 1. Migraine and triggered by angiography (in these cases, the patient should have both diagnoses: the appropriate type or subtype of 1. Migraine and 6.7.2 Angiography headache)

Based on the above points, in order to further discuss the characteristics and potential mechanisms of cerebral AH and improve the applicability of cerebral AH diagnostic criteria in ICHD-3, we designed this prospective study.

## Methods

This project was approved by the Research Ethics Committee of the Chinese People’s Liberation Army (PLA) General Hospital. All of the patients provided written informed consent.

The study included three groups from the First Medical Center of PLA General Hospital, respectively patients admitted to the Department of Neurology and the Department of Cardiology from May 2020 to January 2021, patients admitted to the Department of Vascular and Endovascular Surgery from October 2020 to January 2021. The inclusion criteria were: 1. Older than 18 years of age; and 2. Cerebral angiography or coronary intervention or extremities arterial intervention under local anesthesia. The exclusion criteria were as follows: 1. Patients with subarachnoid hemorrhage, patients who presented with secondary headaches due to the reason for DSA or had a headache occurring within 24 h prior to the procedure; 2. Renal failure requiring dialysis; 3. Severe organ failure, unconsciousness or endotracheal intubation; and 4. Patients who underwent intervention under sedative or general anesthesia; 5. Refusal to complete the questionnaire or follow up regarding the questionnaire.

All patients underwent preoperative serum biochemical tests, blood coagulation, electrocardiogram, chest radiographs or chest CT, and fasting for 8 h and drinking abstaining for 4 h before the procedure. All procedures were performed by senior interventional specialist with the assistance of residents. Cerebral angiography used one or two of Iodoxanol, Ultravist, loversol, lohexol and lopamidol as contrast agents. Coronary intervention includes coronary angiography (CAG) and percutaneous coronary intervention (PCI). Iodixanol or Ultravist was used as contrast agent, and a small amount of nitroglycerin was used during coronary intervention. Extremities arterial intervention consisted of extremities arteriography, balloon dilation, stent implantation, and peripheral aneurysm repair, using iodixanol as contrast agent.

A detailed headache questionnaire was designed by our team based on the ICHD-3 diagnostic criteria. The questionnaire consists of three parts: 1. Basic demographic information and medical history of the patient. 2. Information of interventional procedure. 3. Headache information: (a) Preoperative visit (information of previous headache history): headache age, attack frequency, duration, headache location, quality, intensity, accompanying symptoms, alleviating factors, family headache history, treatment, etc. (b) Postoperative visit (postoperative follow-up time points): headache occurrence, onset time and duration of headache, headache location, quality, intensity, accompanying symptoms, treatment, etc. Intensity was assessed with a numeric rating scale, which is a segmented numeric version of the visual analogue scale (VAS), in which the patients select a whole number from 0 to 10 that best reflects their pain level [11].

### Investigation method

1. Demographic information and related medical history such as: age, gender, body mass index (BMI), education level ≥ high school or not, history of tobacco and alcohol, allergy history, history of intervention, history of hypertension, diabetes, hyperlipidemia, cerebral infarction and information of previous headache history were collected before procedure after informed consent was obtained from patients and their family members. 2. Interventional information, such as type of intervention, type and dose of contrast agent, exposure time in X-ray and intervention procedure pathway were collected through electronic medical record system. 3. The patients were investigated immediately and 24 h, 72 h, 1 week and 2 weeks after the procedure. If discharged, patients were telephoned to follow up on their headaches. It is well known that contrast injection frequently causes a temporary sense of heat in the head and face called “flushing” [4]. In the immediate postoperative investigation, all of the patients were informed about the possibility of “flushing” after the injection of contrast media and were instructed to differentiate this phenomenon from a headache. If a patient with a previous headache reported a headache attack, the characteristics of headache were retrospectively analyzed by two independent neurologists to determine whether the headache type was similar or different from the previous headache. If the two neurologists have different opinions on the diagnosis, a third neurologist will make the judgment.

### Statistical analysis

Statistics were analyzed with the help of IBM SPSS statistics software, version 25.0. Continuous data consistent with normal distribution are reported as the mean ± standard deviation (SD). Hypotheses on differences in means between groups were tested by the independent samples *t* test or and analysis of variance. Median and interquartile range were used to represent continuous data that did not conform to normal distribution, and rank sum test was used for comparison between groups. Categorical data were reported as numbers and percentages. Relationships between categorical variables were analyzed by Fisher’s exact test or Pearson’s chi-square test. Multiple logistic regression analyses were performed on variables that considered significant clinically and with a *p* value < 0.1 on univariate analysis. Using a two-tailed testing, *p* < 0.05 was considered statistically significant.

## Results

### Patients enrollment

**Fig. 1:**
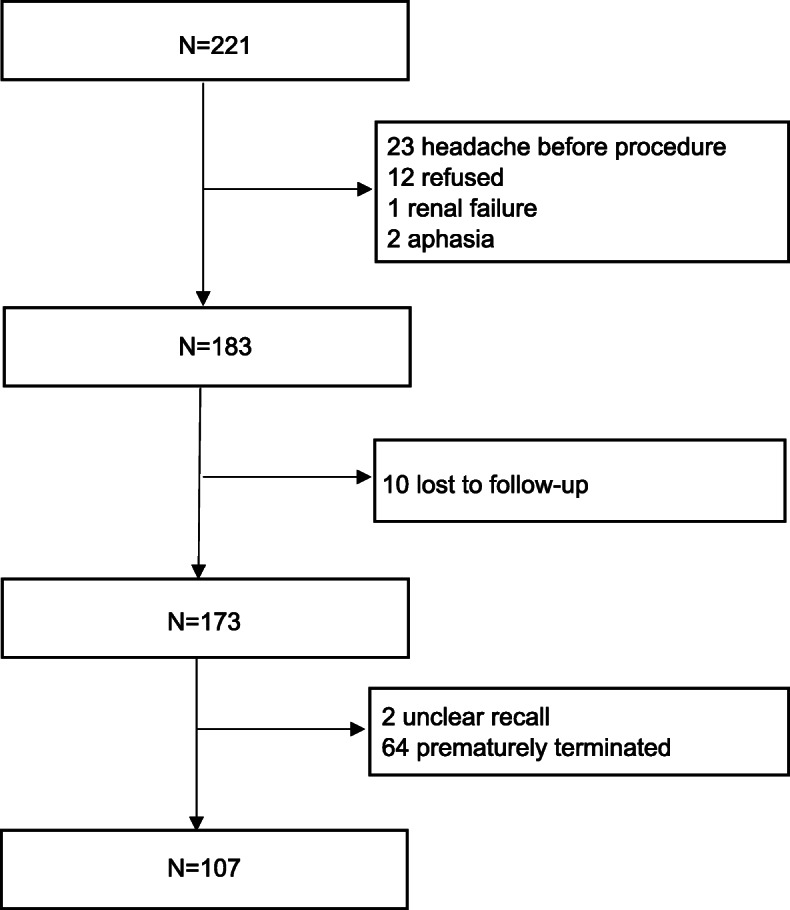
Enrollment of cerebral angiography group

Cerebral angiography group- In total, cerebral angiography was performed in 221 consecutive patients. Twenty-three had headache before interventional procedure, 12 refused to participate in the investigation, 1 was excluded due to renal failure, and 2 patients were unable to cooperate due to aphasia. A total of 183 patients were included. During the 2-week follow-up, 10 patients were lost to follow-up, 2 patients complained of unclear recall, and 64 patients were prematurely terminated due to intracranial stent, aneurysm embolization, or surgical procedures. Ultimately, 107 patients (all diagnostic cerebral angiography) completed the 2-week follow-up (Fig. 1).

**Fig. 2:**
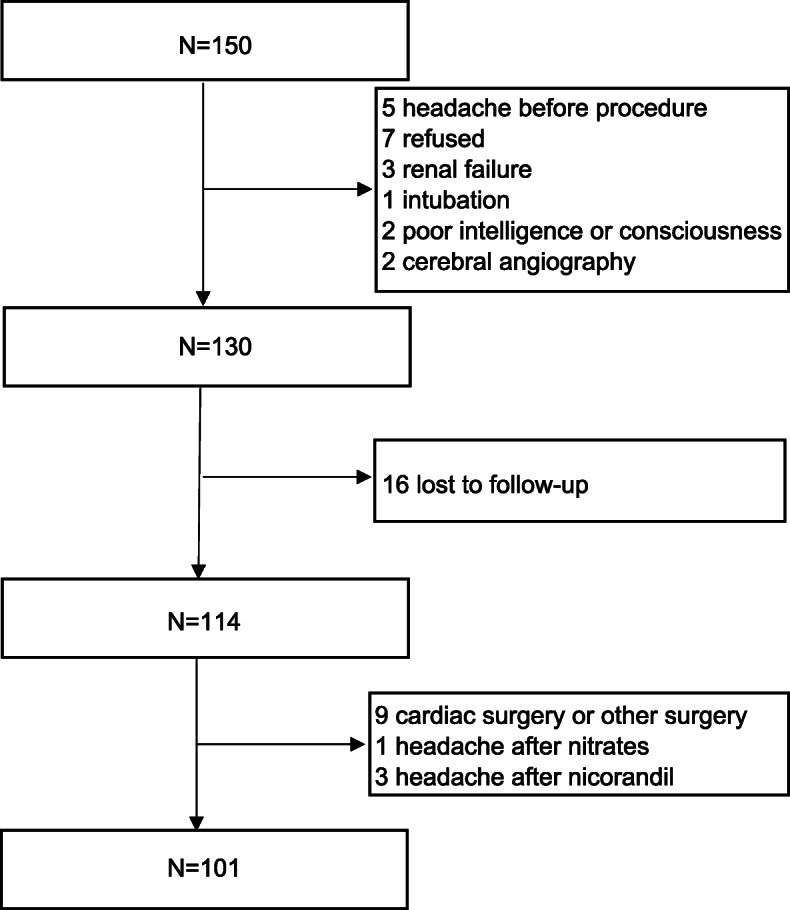
Enrollment of coronary intervention group

Coronary intervention group- Coronary intervention was performed in 150 consecutive patients. Five had headache before interventional surgery, 7 refused to participate in the investigation, 3 were excluded due to renal failure, 1 was unable to participate in the investigation after intubation, 2 could not cooperate with the questionnaire due to poor intelligence or consciousness level and 2 patients underwent coronary angiography and cerebral angiography at the same procedure were excluded. A total of 130 patients were included. During the 2-week follow-up, 16 patients were lost to follow-up, 9 patients were terminated follow-up prematurely for cardiac surgery or other surgery. During the 2-week follow-up, 3 patients had postoperative headaches after the use of nicorandil and 1 had postoperative headache after the use of nitrates. Since most patients with angina and myocardial infarction require vasodilators to dilate the coronary arteries, which can also dilate the intracranial vasculature and cause headaches, during follow up, if patients reported that each headache attack was followed by the vasodilator, and the headache relieved when the vasodilator was discontinued, we believed that the headache was directly caused by the vasodilators, rather than by the procedure. In our study, for these 4 patients, the occurrence of headache exhibited a significantly time-dependent association with the use of nicorandil or nitrates, and headache occurred after each dose of nicorandil or nitrates. All four patients complained of medication-related headaches and refused to take these medications, and the headaches disappeared soon after stopped taking them. Since other patients who regularly took nicorandil or nitrates for a long period of time did not experience this time - and dose-dependent vasodilator - associated headache, we believe that the headache in these 4 patients was caused by nicorandil or nitrates, while the other patients were not. Therefore, these four patients were not included in the analysis. There were 117 procedures performed on 101 patients, including 14 patients who underwent two procedures and 1 underwent three procedures. Only the last procedure was included in the analysis. Ultimately, 101 patients (28 diagnostic coronary angiography and 73 interventional therapy) completed the 2-week follow-up (Fig. 2).

**Fig. 3:**
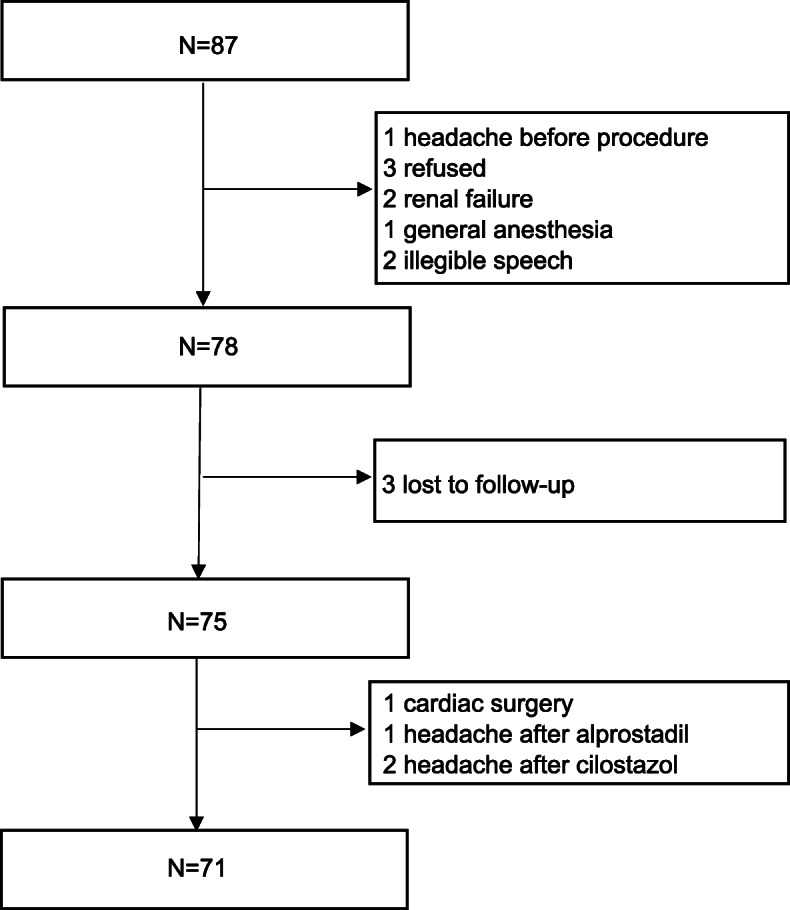
Enrollment of extremities arterial intervention group

Extremities arterial intervention group- Extremities arterial intervention was performed in 87 consecutive patients. One had headache before the procedure, 3 refused to participate in the investigation, 2 were excluded due to renal failure, 1 underwent the procedure under general anesthesia and 2 could not cooperate with the questionnaire survey due to illegible speech. A total of 78 patients were included. During the 2-week follow-up, 3 patients were lost to follow-up, 1 patient was terminated follow-up prematurely for cardiac surgery, 1 patient’s headache was closely related to intravenous alprostadil infusion and 2 patient’s headaches were closely related to cilostazol. There were 79 procedures performed on 71 patients, including 8 patients who underwent two procedures. Only the last procedure was included in the analysis. Ultimately, 71 patients (2 diagnostic angiography and 69 interventional therapy) completed the 2-week follow-up (Fig. 3). Of the 71 patients, 69 underwent lower extremity arterial intervention and 2 underwent upper extremity arterial intervention including 1 subclavian artery occlusion and 1axillary artery stenosis.

### Demographic information

The mean age of the 107 patients in cerebral angiography group was (58.93 ± 9.88) years old, 61.7% (66/107) were male and 38.3% (41/107) were female. The mean length of hospital stay was (9.29 ± 6.73) days, with a median of 7 days. The mean length of hospital stay after cerebral angiography was (4.88 ± 5.10) days, with a median of 3 days. Of the 101 patients in coronary intervention group, 71.3% (72/101) were male, 28.7% (29/101) were female, and their mean age was (62.45 ± 12.79) years old. The mean length of hospital stay was (11.08 ± 7.08) days with a median of 9 days, and the mean length of hospital stay after procedure was (4.31 ± 4.72) days with a median of 3 days. Of the 71 patients in extremities arterial intervention group, 77.5% (55/71) were male, 28.7% (16/71) were female, and their mean age was (66.52 ± 10.77) years old. The mean length of hospital stay was (7.86 ± 2.71) days with a median of 8 days, and the mean length of hospital stay after procedure was (2.75 ± 1.04) days with a median of 2 days.

### Incidence of headache

Twenty-four of 107 (22.4%) patients in cerebral angiography group developed headache within two weeks. The incidence of headache was 7.5% (8/107) during, 6.5% (7/107) within 24 h and 12.1% (13/107) within 2–14 days respectively after the procedure in cerebral angiography group. Of the 24 patients with headache, 29.2% (7/24) were male, 70.8% (17/24) were female and 41.7% (10/24) of patients had more than one headache attack. Twenty-four of 101 (23.8%) patients in coronary intervention group developed headache within two weeks. The incidence of headache was 6.9% (7/101) during, 6.9% (7/101) within 24 h and 14.9% (15/101) within 2–14 days respectively after the procedure in coronary intervention group. Of the 24 patients with coronary intervention related headache, 58.3% (14/24) were male, 41.7% (10/24) were female and 41.7% (10/24) of patients had more than one headache attack. Twelve of 71 (16.9%) patients in extremities arterial intervention group developed headache within two weeks. The incidence of headache was 8.5% (6/71) within 24 h and 11.3% (8/71) within 2–14 days respectively after the procedure in extremities arterial intervention group. Of the 12 patients with extremities arterial intervention related headache, 66.7% (8/12) were male, 33.3% (4/12) were female and 25.0% (3/12) of patients had more than one headache attack. There was no statistical significance in the incidence of headache among the three groups (*p* = 0.535) (Table 2).

**Table 2:** Incidence of headache in three groups

	Cerebral AG	Coronary intervention	EAI	p
Incidence	22.4%	23.8%	16.9%	0.535
HA during the procedure	7.5%	6.9%	N/A	0.067
HA 24 h after the procedure	6.5%	6.9%	8.5%	0.884
HA 2-14 days after the procedure	12.1%	14.9%	11.3%	0.754

*AG* Angiography, *EAI* Extremities arterial intervention, *HA* Headache, *N/A* Not applicable

### History of primary headache in patients

Cerebral angiography group- Among the 107 patients in cerebral angiography group, 26.2% (28/107) patients had a history of headache, including 16 migraine, 6 tension-type headache (TTH) and 6 other types of headache. Of the 24 patients who developed angiography headache, 14 had a history of headache (8 migraine, 3 TTH and 3 other types of headache). Of the 8 patients with headache during angiography, 4 had a history of headache (2 migraine and 2 other types of headache), but the characteristics of headache in 75% (3/4) patients were different from their previous headache. Within the first 24 h after the procedure, headache was seen in 7 patients. Five of the 7 patients had a history of headache (4 migraine and 1 TTH), but the headaches in 60% (3/5) patients were different from their previous headaches. Between days 2–14 after angiography, headache was seen in 13 patients, of which 7 had a history of headache (4 migraine, 2 TTH and 1 other type of headache) and 29% (2/7) patients had different headache characteristics from their previous headaches (Table 3).

**Table 3:** History of primary headache in patients

	Total	No history of HA	History of HA	M	TTH	Others	HA different from previous HA
Cerebral AG							
No HA	83	69	14	8	3	3	N/A
HA	24	10	14	8	3	3	7
HA during	8	4	4	2	0	2	3 (75%)
HA 24 h after	7	2	5	4	1	0	3 (60%)
HA 2–14 days after	13	6	7	4	2	1	2 (29%)
Coronary intervention							
No HA	77	72	5	4	1	0	N/A
HA	24	17	7	3	3	1	2
HA during	7	7	0	0	0	0	N/A
HA 24 h after	7	4	3	1	1	1	1 (33%)
HA 2–14 days after	15	10	5	3	2	0	1 (20%)
EAI							
No HA	59	55	4	0	2	2	N/A
HA	12	8	4	2	1	1	1
HA during	0	0	0	0	0	0	N/A
HA 24 h after	6	3	3	2	1	0	1 (33%)
HA 2–14 days after	8	6	2	1	0	1	0

*HA* Headache, *M* Migraine, *TTH* Tension-type headache, *Others* Other types of headaches, *AG* Angiography, *EAI* Extremities arterial intervention, *N/A* Not applicable

Coronary intervention group- Among the 101 patients in coronary intervention group, 11.9% (12/101) patients had a history of headache, including 7 migraine, 4 TTH and 1 other type headache. Among the 24 patients with coronary intervention headache, 7 patients had a history of headache, including 3 migraine, 3 TTH, and 1 other types of headache. Headache occurred in 7 patients during coronary intervention and none of the 7 patients had a history of headache. Within the first 24 h after coronary intervention, headache was seen in 7 patients. Three of the 7 patients had a history of headache (1 migraine, 1 TTH and 1 other type of headache), but the headache in 1 (33%) patient with other type of headache was different from the previous headache. Between days 2–14 after the procedure, headache was seen in 15 patients, of which 5 had a history of headache (3 migraine and 2 TTH), and 1 (20%) patient with migraine had different headache characteristics from the previous headache (Table 3).

Extremities arterial intervention group- Of the 71 patients in extremities arterial intervention group, 11.3% (8/71) had a history of headache (2 migraine, 3 TTH and 3 other types of headache). Of the 12 patients who developed headache after the procedure, 4 had a history of headache (2 migraine, 1 TTH, and 1 other type headache). None of the patients had headache during the procedure. Within the first 24 h after the procedure, headache was seen in 6 patients. Three of the 6 patients had a history of headache (2 migraine and 1 TTH), and 1 (33%) patient with a history of TTH had different headache characteristics from the previous headache. From 2 to 14 days after the procedure, 8 patients developed headache, 2 of whom had a history of headache (1 migraine and 1 other type of headache), and their headache were similar to the previous headache (Table 3).

### Headache during the procedure

Headache that occurred during cerebral angiography started at a mean time of (36.25 ± 20.13) min after the beginning of the angiography, lasted for an average of (8.27 ± 11.20) min, and the median VAS was 4. The headache occurred on unilateral sides (75.0%), on bilateral sides (25.0%), in the frontal region (25.0%), temporal region (50.0%), occipital region (25.0%), and parietal region (37.5%) with pain associated with throbbing (75.0%), burning (12.5%) or other pain (12.5%) and accompanied by visual aura (37.5%) (Table 4).

**Table 4:** Characteristics of headache during the procedure

	HA during Cerebral AG	HA during Coronary intervention	HA during EAI
	*N* = 8	*N* = 7	*N* = 0
Starting time (min)			
Mean	36.25±20.13	15.40±13.63	0
Median	37.50	10	0
IQR	41	27	0
Duration (min)			
Mean	8.27±11.20	24.71±19.51	0
Median	3	30	0
IQR	17.42	25	0
Position			
Unilateral	6 (75.0%)	2 (28.6%)	0
Bilateral	2 (25.0%)	5 (71.4%)	0
Location			
Frontal	2 (25.0%)	1 (14.3%)	0
Temporal	4 (50.0%)	4 (57.1%)	0
Occipital	2 (25.0%)	1 (14.3%)	0
Parietal	3 (37.5%)	2 (28.6%)	0
Full head	0	2 (28.6%)	0
Intensity (VAS)			
Mean	4.13±2.64	4.86±2.27	0
Median	4	4	0
IQR	4	4	0
Quality			
Throbbing	6 (75.0%)	5 (71.4%)	0
Pressing	0	2 (28.6%)	0
Stabbing	0	0	0
Burning	1 (12.5%)	0	0
Others	1 (12.5%)	0	0
Associated symptoms			
Nausea	0	1 (14.3%)	0
Vomiting	0	0	0
Photophobia	0	2 (28.6%)	0
Phonophobia	0	2 (28.6%)	0
Aura	3 (37.5%)	2 (28.6%)	0

*AG* Angiography, *EAI* Extremities arterial intervention, *HA* Headache, *IQR* Interquartile range, *VAS* Visual analogue scale

Headache that occurred during the coronary intervention started at a mean time of (15.4 ± 13.63) min after the beginning of the intervention, lasted for an average of (24.71 ± 19.51) min, and the median VAS was 4. The headache occurred on unilateral sides (28.6%), on bilateral sides (71.4%), in the frontal region (14.3%), temporal region (57.1%), occipital region (14.3%), parietal region (28.6%), full head (28.6%) with pain associated with throbbing (71.4%), pressing (28.6%) and accompanied by nausea (14.3%), photo (28.6%) - or phonophobia (28.6%) or visual aura (28.6%) (Table 4).

No headache occurred during the procedure in extremities arterial intervention group. The incidence of headache during the procedure was 7.5% (8/107) in cerebral angiography group and 6.9% (7/101) in coronary intervention group. There was no statistical significance in the incidence of headache during the procedure between the two groups (*p* = 0.067) (Table 2). Compared with coronary intervention group, the onset time of headache during cerebral angiography was later and the duration of headache was shorter, but statistical analysis showed that there was no statistical significance in headache onset time (*p* = 0.068), duration (*p* = 0.054) and VAS (*p* = 0.613) in the two groups (Table 5).

**Table 5:** Comparison of characteristics of headache during the procedure

	Cerebral AG	Coronary intervention	p
Starting time (min)			
Mean ± SD	36.25±20.13	15.40±13.63	0.068
Duration (min)			
Median	3	30	0.054
IQR	17.42	25	
VAS			
Median	4	4	0.613
IQR	4	4	

*AG* Angiography, *SD* Standard deviation, *IQR* Interquartile range, *VAS* Visual analogue scale

### Headache within 24 h after the procedure

Headache occurring within 24 h after completion of cerebral angiography started at a mean time of (7.69 ± 7.86) h and the median onset time was 4.5 h, lasted for an average of (400.00 ± 363.90) min and the median duration was 420 min, and the median VAS was 3. The headache occurred on unilateral sides (28.6%), on bilateral sides (71.4%), in the frontal region (71.4%), temporal region (14.3%), parietal region (14.3%), full head (14.3%) and with pain associated with throbbing (57.1%), pressing (14.3%) stabbing (14.3%) or others (14.3%) and accompanied by photo (14.3%) - or phonophobia (14.3%) or visual aura (28.6%) (Table 6).

**Table 6:** Characteristics of headache 24 h after the procedure

	HA 24 h after Cerebral AG	HA 24 h after Coronary intervention	HA 24 h after EAI
	*N* = 7	*N* = 7	*N* = 6
Starting time (h)			
Mean	7.69±7.86	4.14±2.11	10.75±7.24
Median	4.5	4	7.75
IQR	12.3	2	12.8
Duration (min)			
Mean	400.00±363.90	789.29±715.27	2170.00±3900.91
Median	420	720	750
IQR	714	960	3360
Position			
Unilateral	2 (28.6%)	1 (14.3%)	1 (16.7%)
Bilateral	5 (71.4%)	6 (85.7%)	5 (83.3%)
Location			
Frontal	5 (71.4%)	2 (28.6%)	2 (33.3%)
Temporal	1 (14.3%)	5 (71.4%)	1 (16.7%)
Occipital	0	0	0
Parietal	1 (14.3%)	1 (14.3%)	3 (50.0%)
Full head	1 (14.3%)	0	1 (16.7%)
Intensity (VAS)			
Mean	3.38±1.51	4.43±1.51	4.83±1.60
Median	3	4	5.5
IQR	3	3	3
Quality			
Throbbing	4 (57.1%)	5 (71.4%)	4 (66.7%)
Pressing	1 (14.3%)	1 (14.3%)	1 (16.7%)
Stabbing	1 (14.3%)	0	0
Burning	0	0	1 (16.7%)
Others	1 (14.3%)	1 (14.3%)	1 (16.7%)
Associated symptoms			
Nausea	0	2 (28.6%)	2 (33.3%)
Vomiting	0	0	0
Photophobia	1 (14.3%)	1 (14.3%)	2 (33.3%)
Phonophobia	1 (14.3%)	2 (28.6%)	2 (33.3%)
Aura	2 (28.6%)	1 (14.3%)	1 (16.7%)

*AG* Angiography, *EAI* Extremities arterial intervention, *HA* Headache, *IQR* Interquartile range, *VAS* Visual analogue scale

Headache occurring within 24 h after completion of coronary intervention started at a mean time of (4.14 ± 2.11) h and the median onset time was 4 h, lasted for an average of (789.29 ± 715.27) min and the median duration was 720 min, and the median VAS was 4. The headache occurred on unilateral sides (14.3%), on bilateral sides (85.7%), in the frontal region (28.6%), temporal region (71.4%), parietal region (14.3%) and with pain associated with throbbing (71.4%), pressing (14.3%), others (14.3%) and accompanied by nausea (28.6%), photo (14.3%) - or phonophobia (28.6%) or visual aura (14.3%) (Table 6).

Headache occurring within 24 h after completion of extremities arterial intervention started at a mean time of (10.75 ± 7.24) h and the median onset time was 7.75 h, lasted for an average of (2170.00 ± 3900.91) min and the median duration was 750 min, and the median VAS was 5.5. The headache occurred on unilateral sides (16.7%), on bilateral sides (83.3%), in the frontal region (33.3%), temporal region (16.7%), parietal region (50.0%), full head (16.7%) and with pain associated with throbbing (66.7%), pressing (16.7%), burning (16.7%), others pain (16.7%) and accompanied by nausea (33.3%), photo (33.3%) - or phonophobia (33.3%) or visual aura (16.7%) (Table 6).

The incidence of headache within 24 h after the procedure in the three groups was 6.5% (7/107), 6.9% (7/101) and 8.5% (6/71) respectively in cerebral angiography group, coronary intervention group and extremities arterial intervention group and statistical results showed that it was no statistically significant (*p* = 0.884) (Table 2). Statistical analysis showed that there was no statistical significance in headache onset time (*p* = 0.097), duration (*p* = 0.328) and VAS (*p* = 0.206) in the three groups (Table 7).

**Table 7:** Comparison of characteristics of headache 24 h after the procedure

	Cerebral AG	Coronary intervention	EAI	p
Starting time (h)				
Median	4.5	4	7.75	0.097
IQR	12.3	2	12.8	
Duration (min)				
Median	420	720	750	0.328
IQR	714	960	3360	
VAS				
(Mean ± SD)	3.38±1.51	4.43±1.51	4.83±1.60	0.206

### Headache within 2–14 days after the procedure

The mean onset time of headache within 2–14 days after completion of cerebral angiography started at a mean time of (6.30 ± 4.02) days and the median onset time was 5 days, lasted for an average of (123.72 ± 200.77) min and the median duration was 30 min, and the median VAS was 3. The headache occurred on unilateral sides (53.8%), on bilateral sides (46.2%), in the frontal region (30.8%), temporal region (38.5%), occipital region (23.1%), parietal region (53.8%), and with pain associated with throbbing (69.2%), pressing (23.1%), stabbing (15.4%) and accompanied by nausea (7.7%), photo (7.7%) - or phonophobia (15.4%) (Table 8).

**Table 8:** Characteristics of headache 2-14 days after the procedure

	HA 2-14 days after Cerebral AG (*N* = 13)	HA 2-14 days after Coronary intervention (*N* = 15)	HA 2-14 days after EAI (*N* = 8)
Starting time (days)			
Mean	6.30±4.02	6.89±4.04	6.09±2.81
Median	5	7	6
IQR	7	8	5
Duration (min)			
Mean	123.72±200.77	412.93±760.60	228.00±439.52
Median	30	60	60
IQR	119.92	468	215
Position			
Unilateral	7 (53.8 %)	8 (53.3 %)	5 (62.5 %)
Bilateral	6 (46.2%)	7 (46.7%)	3 (37.5%)
Location			
Frontal	4 (30.8 %)	4 (26.7 %)	0
Temporal	5 (38.5%)	7 (46.7%)	4 (50.0%)
Occipital	3 (23.1%)	2 (13.3%)	2 (25.0%)
Parietal	7 (53.8%)	3 (20.0%)	1 (12.5%)
Full head	0	1 (6.7%)	1 (12.5%)
Intensity (VAS)			
Mean	3.43±1.47	3.61±1.29	3.27±1.35
Median	3	3	4
IQR	2	2	1
Quality			
Throbbing	9 (69.2%)	10 (66.7%)	7 (87.5%)
Pressing	3 (23.1%)	1 (6.7%)	0
Stabbing	2 (15.4%)	1 (6.7%)	1 (12.5%)
Burning	0	2 (13.3%)	0
Others	0	1 (6.7%)	0
Associated symptoms			
Nausea	1 (7.7%)	1 (6.7%)	0
Vomiting	0	0	0
Photophobia	1 (7.7%)	2 (13.3%)	0
Phonophobia	2 (15.4%)	4 (26.7%)	1 (12.5%)
Aura	0	1 (6.7%)	1 (12.5%)

*AG* Angiography, *EAI* Extremities arterial intervention, *HA* Headache, *IQR* Interquartile range, *VAS* Visual analogue scale

The mean onset time of headache within 2–14 days after completion of coronary intervention started at a mean time of (6.89 ± 4.04) days and the median onset time was 7 days, lasted for an average of (412.93 ± 760.60) min and the median duration was 60 min, and the median VAS was 3. The headache occurred on unilateral sides (53.3%), on bilateral sides (46.7%), in the frontal region (26.7%), temporal region (46.7%), occipital region (13.3%), parietal region (20.0%), full head (6.7%) with pain associated with throbbing (66.7%), pressing (6.7%), stabbing (6.7%), burning (13.3%), others (6.7%) and accompanied by nausea (6.7%), photo (13.3%) - or phonophobia (26.7%) or visual aura (6.7%) (Table 8).

The mean onset time of headache within 2–14 days after completion of extremities arterial intervention started at a mean time of (6.09 ± 2.81) days and the median onset time was 6 days, lasted for an average of (228.00 ± 439.52) min and the median duration was 60 min, and the median VAS was 4. The headache occurred on unilateral sides (62.5%), on bilateral sides (37.5%), in the temporal region (50.0%), occipital region (25.0%), parietal region (12.5%), full head (12.5%) and with pain associated with throbbing (87.5%), stabbing (12.5%) and accompanied by phonophobia (12.5%) or visual aura (12.5%) (Table 8).

The incidence of headache in the three groups was 12.1% (13/107), 14.9% (15/101) and 11.3% (8/71) within 2 to 14 days after the procedure and it was also no statistical significance (*p* = 0.754) (Table 2). Statistical analysis showed that there was no statistical significance in headache onset time (*p* = 0.816), duration time (*p* = 0.129) and VAS (*p* = 0.771) in the three groups (Table 9).

**Table 9:** Comparison of characteristics of headache 2-14 days after the procedure

	Cerebral AG	Coronary intervention	EAI	p
Starting time (day)				
Median	5	7	6	0.816
IQR	7	8	5	
Duration (min)				
Median	30	60	60	0.129
IQR	119.92	468	215	
VAS				
Median	3	3	4	0.771
IQR	2	2	1	

*AG* Angiography, *EAI* Extremities arterial intervention, *VAS* Visual analogue scale, *IQR* Interquartile range

### Potential risk factors related to headache

Patient demographics of cerebral angiography (*n* = 107) were shown in Table 10. There were significant differences in sex, smoking history and previous history of headache between the no headache group and the headache group by univariate analysis (*p*<0.001, *p* = 0.006 and *p*<0.001). No statistical differences were found in age (*p* = 0.395), BMI (*p* = 0.217), education (*p* = 0.266), alcohol use (*p* = 0.132), hypertension (*p* = 0.092), diabetes (*p* = 0.707), cerebral infarction history (*p* = 0.350), hyperlipidaemia (*p* = 1.00) and coronary heart disease (*p* = 0.184), whether there was cerebral vascular stenosis (*p* = 0.407), cerebral aneurysm(*p* = 0.560) or current cerebral infarction (*p* = 0.149), previous history of intervention (*p* = 0.407), transfemoral or transradial pathway (*p* = 0.242) in the headache and no-headache groups.

**Table 10:** Potential risk factors related to cerebral angiography headache in univariate analysis

Variables	Total (*N* = 107)	No HA (*N* = 83)	HA (*N* = 24)	P
Sex				
Male	66 (61.7%)	59 (71.1%)	7 (29.2%)	<0.001
Female	41 (38.3%)	24 (28.9%)	17 (70.8%)	
Age (years)	58.93±9.88	59.37±10.18	57.42±8.79	0.395
BMI (kg/m^2^)	25.60±5.34	25.95±5.88	24.41±2.53	0.217
Education				
≥High school	64 (59.8%)	52 (62.7%)	12 (50.0%)	0.266
<High school	43 (40.2%)	31 (37.3%)	12 (50.0%)	
History of allergy	14 (13.1%)	9 (10.8%)	5 (20.8%)	0.299
Current smoking status	44 (41.1%)	40 (48.2%)	4 (16.7%)	0.006
Current alcohol use	36 (33.6%)	31 (37.3%)	5 (20.8%)	0.132
History of headache	28 (26.2%)	14 (16.9%)	14 (58.3%)	<0.001
Hypertension	69 (64.4%)	57 (68.7%)	12 (50.0%)	0.092
Diabetes	30 (28.0%)	24 (28.9%)	6 (8.3%)	0.707
History of cerebral infarction	17 (15.9%)	15 (18.1%)	2 (8.3%)	0.350
Hyperlipaemia	9 (8.4%)	7 (8.4%)	2 (8.3%)	1.000
Coronary heart disease	14 (13.1%)	13 (15.7%)	1 (4.2%)	0.184
History of intervention	50 (46.7%)	37 (44.6%)	13 (54.2%)	0.407
Pathway				
Transradial	21 (19.6%)	14 (16.9%)	7 (29.2%)	0.242
Transfemoral	86 (80.4%)	69 (83.1 %)	17 (70.8%)	
Vascular stenosis				
<Severe	57 (53.3%)	46 (55.4%)	11 (45.8%)	0.407
≥Severe	50 (46.7%)	37 (44.6%)	13 (54.2%)	
Cerebral aneurysm	21 (19.6%)	15 (18.1%)	6 (25.0%)	0.560
Current cerebral infarction	22 (20.6%)	20 (24.1%)	2 (8.3%)	0.149

*HA* Headache, *BMI* Body mass index

Patient demographics of coronary intervention (*n* = 101) are shown in Table 11. Patients who had a history of primary headache were more likely to have coronary intervention headache by univariate analysis (*p* = 0.007). No statistical differences were found in sex (*p* = 0.108), age (*p* = 0.192), BMI (*p* = 0.142), education (*p* = 0.205), allergy history (*p* = 0.292), smoking history (*p* = 0.565), alcohol history (*p* = 0.175), hypertension (*p* = 0.629), diabetes (*p* = 0.403), cerebral infarction (*p* = 1.000), hyperlipidaemia (*p* = 0.070) history of intervention (*p* = 0.493), transfemoral or transradial pathway (*p* = 0.062), type of contrast agent used (*p* = 0.138), quantity of contrast agent used (*p* = 0.055 and 0.495), X-ray exposure time during intervention (*p* = 0.050), indication for intervention (*p* = 0.804) or type of intervention (*p* = 0.482) in the headache and no-headache groups.

**Table 11:** Potential risk factors for related to coronary intervention headache in univariate analysis

Variables	Total (*N* = 101)	No HA (*N* = 77)	HA (*N* = 24)	P
Sex				
Male	72 (71.3%)	58 (75.3%)	14 (58.3%)	0.108
Female	29 (28.7%)	19 (24.7%)	10 (41.7%)	
Age (years)	62.45±12.79	63.38±13.37	59.46±10.41	0.192
BMI (kg/m^2^)	25.60±5.34	25.84±3.83	26.73±3.24	0.142
Education				
≥high school	56 (55.4%)	40 (51.9%)	16 (66.7%)	0.205
<high school	45 (44.6%)	37 (48.1%)	8 (33.3%)	
History of allergy	13 (12.9%)	8 (10.4%)	5 (20.8%)	0.292
Current smoking status	43 (42.6%)	34 (44.2%)	9 (37.5%)	0.565
Current alcohol use	37 (36.6%)	31 (40.3%)	6 (25.0%)	0.175
History of headache	12 (11.9%)	5 (6.5%)	7 (29.2%)	0.007
Hypertension	59 (58.4%)	46 (59.7%)	13 (54.2%)	0.629
Diabetes	27 (26.7%)	19 (24.7%)	8 (33.3%)	0.403
History of cerebral infarction	8 (7.9%)	6 (7.8%)	2 (8.3%)	1.000
Hyperlipaemia	24 (23.8%)	15 (19.5%)	9 (37.5%)	0.070
History of intervention	44 (43.6%)	35 (45.5%)	9 (37.5%)	0.493
Pathway				
transradial	90 (89.1%)	66 (85.7%)	24 (100%)	0.062
transfemoral	11 (10.9%)	11 (14.3%)	0	
Contrast media				
Iodixanol	54 (53.5%)	38 (49.4%)	16 (66.7%)	0.138
Ultravist	47 (46.5%)	39 (50.6%)	8 (33.3%)	
Contrast media volume (ml)				
Iodixanol	152.78±92.42	167.37±92.87	118.13±84.16	0.055
Ultravist	167.23±98.61	173.59±103.50	136.25±66.53	0.495
Exposure time in X-ray (min)	19.42±13.39	20.40±13.90	16.26±11.32	0.050
Indication				
angina	58 (57.4%)	43 (55.8%)	15 (62.5%)	0.804
AMI	27 (26.7%)	22 (28.6%)	5 (20.8%)	
Others	16 (15.8%)	12 (15.6%)	4 (16.7%)	
Intervention				
CAG	28 (27.7%)	20 (26.0%)	8 (33.3%)	0.482
PCI	73 (72.3%)	57 (74.0%)	16 (66.7%)	

*HA* Headache, *BMI* Body mass index, *AMI* Acute myocardial, *CAG* Coronary angiography, *PCI* Percutaneous coronary intervention

Patient demographics of extremities arterial intervention (*n* = 71) were shown in Table 12. Patients who had a history of primary headaches were more likely to have extremities arterial intervention related headache by univariate analysis (*p* = 0.024). No statistical differences were found in sex (*p* = 0.448), age (*p* = 0.143), BMI(*p* = 0.248), education (*p* = 1.000), allergy history (*p* = 0.213), smoking history (*p* = 0.507), alcohol history (*p* = 0.627), hypertension (*p* = 0.335), diabetes (*p* = 0.370), coronary heart disease (*p* = 0.125), cerebral infarction (*p* = 0.336), hyperlipidaemia (*p* = 1.000) history of intervention (*p* = 0.758), transfemoral or transradial pathway (*p* = 0.581) and quantity of contrast agent used (*p* = 0.416) in the headache and no-headache groups.

**Table 12:** Potential risk factors for headache related to EAI headache in univariate analysis

Variables	Total (*N* = 71)	No HA (*N* = 59)	HA (*N* = 12)	P
Sex				
male	55 (77.5%)	47 (79.7%)	8 (66.7%)	0.448
female	16 (28.7%)	12 (20.3%)	4 (33.3%)	
Age (years)	66.52±10.77	67.19±11.01	63.25±9.22	0.143
BMI (kg/m^2^)	24.63±3.27	24.84±3.06	23.63±4.13	0.248
Education				
≥high school	28 (39.4%)	23 (39.0%)	5 (41.7%)	1.000
<high school	43 (60.6%)	36 (61.0%)	7 (58.3%)	
History of allergy	13 (18.3%)	9 (15.3%)	4 (33.3%)	0.213
Current smoking status	48 (67.6%)	41 (69.5%)	7 (58.3%)	0.507
Current alcohol use	40 (56.3%)	34 (57.6%)	6 (50.0%)	0.627
History of headache	8 (11.3%)	4 (6.8%)	4 (33.3%)	0.024
Hypertension	45 (63.4%)	39 (66.1%)	6 (50.0%)	0.335
Diabetes	32 (45.1%)	28 (47.5%)	4 (33.3%)	0.370
Coronary heart disease	16 (22.5%)	11 (18.6%)	5 (41.7%)	0.125
History of cerebral infarction	8 (11.3%)	8 (13.6%)	0	0.336
Hyperlipaemia	7 (9.9%)	6 (10.2%)	1 (8.3%)	1.000
History of intervention	44 (62.0%)	36 (61.0%)	8 (66.7%)	0.758
Pathway				
transradial	6 (8.5%)	4 (6.8%)	2 (16.7%)	0.581
transfemoral	65 (91.5%)	55 (93.2%)	10 (83.3%)	
Contrast media volume (ml)	102.43±34.07	103.22±35.35	98.18±27.14	0.416

*EAI* Extremities arterial intervention, *HA* Headache, *BMI* Body mass index

Variables with *p* < 0.1 in univariate analysis and variables that considered significant clinically were included in Logistic regression for multivariate analysis. After adjusting for sex, education level, smoking history, history of headache and history of hypertension in Logistic regression model, patients with history of headache had an increased risk of cerebral angiography headache compared with those without history of headache (OR = 4.850; 95% CI, 1.695–13.884; *P* = 0.003), female had an increased risk of cerebral angiography headache (OR = 4.189; 95% CI, 1.451–12.097; *P* = 0.008). In coronary intervention group, after adjusting for sex, education level, history of headache, history of hypertension, hyperlipidemia, interventional pathway and X-ray exposure time in Logistic regression model, patients with history of headache had an increased risk of coronary intervention-related headache compared with those without history of headache (OR = 5.929; 95% CI, 1.676–20.977; *P* = 0.006). In extremities arterial intervention group, after adjusting for sex, education level, history of headache and history of hypertension in Logistic regression model, patients with history of headache had an increased risk of headache related to extremities arterial intervention compared with those without history of headache (OR = 6.875; 95% CI, 1.428–33.107; *P* = 0.016) (Table 13).

**Table 13:** Potential risk factors for headache in Logistic analysis

Variables	OR (95% CI)	P
Cerebral AG		
History of headache		
No	1.000	
Yes	4.850 (1.695-13.884)	0.003
Sex		
Male	1.000	
Female	4.189 (1.451-12.097)	0.008
Coronary intervention		
History of headache		
No	1.000	
Yes	5.929 (1.676-20.977)	0.006
EAI		
History of headache		
No	1.000	
Yes	6.875 (1.428-33.107)	0.016

*OR* Odds ratio, *CI* Confidence interval, *AG* Angiography, *EAI* Extremities arterial intervention

## Discussion

The diagnosis of headache is mainly based on the clinical characteristics of headache, such as headache onset pattern, frequency, duration, location, quality, intensity, accompanying symptoms and alleviating factors. In ICHD-3, the diagnostic criteria for *6.7.2 angiography headache* do not describe the location, quality, intensity and accompanying symptoms of headache (Table 1). In ICHD-3 beta version, the diagnostic criteria for angiography headache (Chapter 6.7.2) have only been based on three studies [12].The diagnostic criteria for angiography headache in ICHD-3 were based on five studies, only one study included peripheral angiography as a control group [13].

Of the 5 studies, all patients included in Shuaib et al. ‘s study had a history of migraine, and their study focused on the relationship between migraine and neurological complications after angiography [14]. The study of Ramadan et al. only included 45 patients, with a small sample size and a short follow-up time [2]. The study of Gil-Gouveia included 32 patients with subarachnoid hemorrhage, and only 48 patients after subarachnoid hemorrhage was removed [1]. Gündüz’s study mainly focused on headaches related to angiographic therapeutic procedures [3]. The study of Aktan was a prospective case-control study, including 139 cases of cerebral angiography and 30 cases of peripheral angiography. However, the sample size of the control group in the study was only 30 cases, which was significantly less than that of the experimental group. Therefore, the incidence of headache in the two groups could not be statistically analyzed, and the characteristics of headache in the control group were not described. In addition, in Aktan ‘s study, patients were informed before angiography of the possibility of headache during and after angiography, which may bring mental stress and thus affect the incidence of headache [4].

Our study showed that the incidence of headache in cerebral angiography was 22.4% (24/107), 23.8% (24/101) in coronary intervention and 16.9% (12/71) in extremities arterial intervention. The incidence of headache in the cerebral angiography and coronary intervention group was similar, slightly higher than that in extremities arterial intervention group, and there was no statistical significance in the incidence of headache in the three groups.

In our study, the incidence of headache in coronary intervention group (23.8%, 24/101) and extremities arterial intervention group (16.9%, 12/71) was higher than the incidence of headache in peripheral angiography group of Aktan (10%, 3/30) [4]. This may be due to that the control group only had 30 patients, which could easily cause bias. There were a few studies on cerebral angiography headache, and the incidence rate was different in the previous reports, which may be caused by different studies had different study methods. In terms of research methods, for a prospective cohort study, we set three groups: cerebral angiography group, coronary intervention group and extremities arterial intervention group. In order to reduce patient recall bias, we set five follow-up time points: immediately after the procedure, 24 h, 72 h, 1 week and 2 weeks after the procedure. During the exclusion criteria and follow-up, we excluded factors which might influence the incidence of headache, for example, not informing patients of the possibility of headache related to the intervention before the procedure, avoiding psychological suggestion to patients, and thus reducing headache caused by mental factors such as anxiety [15, 16]. We excluded patients had headache within 24 h prior to the procedure in order to avoid interference with the procedure related headache. Patients with renal failure requiring dialysis may have *10.2 dialysis headache* and were excluded [13, 17]. The above experimental scheme design improved the quality control of our study.

In our study, female was not a risk factor for headache in coronary intervention group and extremities arterial intervention group, but a risk factor for cerebral angiography headache, which is consistent with the research of Gil-Gouveia [1]. The possible explanation is that women demonstrating lower pain thresholds and the fluctuations of sex hormones especially estrogen and progesterone may cause a change in the prevalence or intensity of headache [1, 18, 19]. Previous history of headache as a risk factor for angiography headache had been established in many studies [2–6, 20]. The possible mechanism maybe that some individuals with a history of headache had lower thresholds for trigeminal activation [2, 7]. Although previous literature had reported that patients with cerebral aneurysms or cerebral infarction were prone to headache [13, 21], our study and previous studies showed that the occurrence of cerebral angiography headache was not related to these angiographic indications [2, 3]. High education level was a risk factor for angiography headache in study of Kwon. They suggested that it was possible that patients with high education level were more anxious and/or nervous with the procedure [5]. In our study, beyond high school was not a risk factor for cerebral angiography headache, possibly because education level could not represent mental state. Future researches should study education level together with related mental scales such as anxiety and depression. We studied the incidence of angiography headache of the two procedure approaches namely transradial and transfemoral, the results showed that there was no statistical significance between the two procedure approaches. Other factors such as age, BMI, allergic history, tobacco and alcohol history, hypertension, diabetes, cerebral infarction, hyperlipidemia, coronary heart disease, intervention history, and whether complicated with severe vascular stenosis were not risk factors for cerebral angiography headache.

Previous study had shown that the occurrence of migraine after cerebral angiography was related to meningeal vasodilation [9]. Studies had reported that patients underwent cerebrovascular interventional therapy had a higher incidence of headache than cerebral angiography [3, 7]. The occurrence of headache during cranial embolization has been attributed to vessel distension occurring with balloon inflation, which stimulates pain receptors in the vessel wall, producing focal areas of referred pain [22–24]. Theoretically, it can also occur with contrast injection, if the pressure within the vessel is enough to reach the threshold of mechanical stimulation of pain receptors [7]. In the cerebral angiography group and coronary intervention group of our study, the intraoperative headache occurred within 1 h after the beginning of the procedure, the duration time was less than 1 h. Most of the patients had mild to moderate throbbing headache, and the headache characteristics were similar to *8.1.1.1 immediate NO donor-induced headache* in ICHD-3 [13, 25, 26]. There were no statistical differences in onset time, duration and VAS of postoperative headache among cerebral angiography group, coronary intervention group and extremities arterial intervention group, and most of the headaches were throbbing, suggesting that the cause of headache might be as hypothesized by Ramadan-the catheter or the contrast material activate platelets or damage the vascular endothelium thereby resulting in the release of vasoactive substances such as nitric oxide (NO) or serotonin (5-HT) which have been implicated in the pathogenesis of vascular headache [2]. In our study extremities arterial intervention group had no intraoperative headache. The probable explanation is that during cerebral angiography and coronary intervention, catheterization and/or contrast agents pass through the brachioid trunk and the aortic arch, damaging and inducing vascular endothelial cells to produce vasoactive substances (such as NO, 5-HT, etc.). These vasoactive substances may enter the brain via the carotid and/or vertebral arteries and cause headache. In our study, 97.2% (69/71) of patients in extremities arterial intervention group underwent lower extremity arterial intervention and 91.5% (65/71) were transfemoral approaches. Catheters and contrast agents mostly did not pass through the brachioid trunk and the aortic arch. In addition, since the lower extremity arteries are relatively far away from the brain, the vasoactive substances produced by the injury-induced vascular endothelial cells during the interventional procedure are rapidly metabolized and absorbed instead of entering the brain. The mechanism of cerebral angiography headache is unknown, and no related animal experiments have been conducted to explore the mechanism. In the future, more studies are needed to pay attention to the mechanism.

**Fig. 4:**
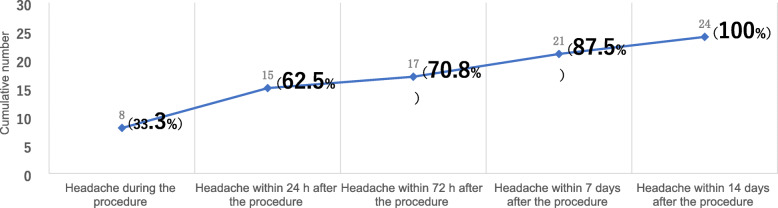
Cumulative frequency distribution of patients with angiography headache(*N* = 24)

In ICHD-3, diagnostic criterion A in 6.7.2 is “Any new headache fulfilling criterion C”. criterion C1 is “headache has developed during or within 24 hours of the angiography”. Both our study and previous reports have confirmed the headache during the procedure [3, 4, 7]. In the study of Aktan [4], of all patients who developed angiography headache, 23.8% (10/42) was during the procedure, 61.9% (26/42) was within 24 h after the procedure and 45.2% (19/42) was within 2–7 days after the procedure. The onset time of headache in these 45.2% patients was not within the time range defined by criterion C1. In our study, headache still occurred in 13 patients within 2–14 days after the procedure, accounting for 54.2% (13/24) of the total headache patients, and these 54.2% patients was also not within the time range defined by criterion C1. Figure 4 shows the cumulative frequency distribution of patients with cerebral AH at each follow-up time point in our study. Patients with cerebral AH during the procedure was 33.3% (8/24), up to 24 h after the procedure, the patients with cerebral AH accounted for 62.5% (15/24) of the total number of patients with cerebral AH, up to 3 days after the procedure and up to 7 days after the procedure, the patients with cerebral AH accounted for 70.8% (17/24) and 87.5% (21/24) respectively. According to our results, C1 criteria only included 62.5% of patients with cerebral AH. If the time specified in the criterion C1 is extended to 1 week after the procedure, 87.5% of patients with headache can be included, which will significantly improve the sensitivity of the criterion C1. Therefore, we suggest that the criterion C1 should be changed to: headache has developed during or within 7 days of the angiography.

Criterion C2 defines as “headache has resolved within 72 hours after the angiography”. In our cerebral angiography group, the average duration of headache during the procedure was (8.27 ± 11.20) min, with a median of 3 min; The average duration of headache within 24 h after the procedure was (400.00 ± 363.90) min, with a median of 420 min. The average duration of headache within 2–14 days after the procedure was (123.72 ± 200.77) min, with a median of 30 min. Aktan reported that the mean duration of headache during angiography was (41.7 ± 65.4) min, with a median of 22.5 min. The mean duration of headache within 24 h after the procedure was (171.7 ± 208.1) min with a median of 75 min, and the mean duration within 2–7 days after the procedure was (122.1 ± 173.1) min with a median time of 60 min [4]. Kwon reported that 77.9% of headaches resolved within 24 h [5]. The criterion C2 is defined as headache resolved within 72 h after the angiography. The time span is obviously too long, so we recommend that criterion C2 be modified to: headache resolved within 24 h of a single headache attack.

Criterion C3a defines as “developing during contrast injection and lasting <1 hour”. In our study, a total of 8 patients developed headache during angiography, with an average duration of (8.27 ± 11.20) min and a median duration of 3 min. Aktan reported [4] that the mean duration of headache during angiography was (41.7 ± 65.4) min, with a median of 22.5 min. Gündüz reported [3] the duration of headache during angiography was less than 10 min. Gil-Gouveia Rreported [7] the duration of headache during angiography varying from 2 to 30 s. These findings support criterion C3a, therefore, it is recommended that criterion C3a should be retained.

Criterion C3b defines as “developing a few hours after the angiography and lasting >24 hours”. The criterion C3b and the criterion C3a are in parallel relationship. The criterion C3a is for the characteristics of headache during angiography, while the criterion C3b is obviously for the characteristics of headache after angiography. As in the previous discussion of criterion C1 and C2, the description of criterion C3b that headache developing a few hours after the angiography and lasting >24 h is obviously not appropriate. It is suggested to be modified to “ developing a few hours to 7 days after angiography and a single headache lasting < 24 hours”.

Criterion C3c defines as “occurring in a patient with *1. Migraine* and having the features of *1.1 Migraine without aura* or *1.2 Migraine with aura*” In our study, some patients with a history of migraine developed angiography headache with the same characteristics as their previous migraine, and some patients had visual aura, which is supported by previous reports [2, 6]. It is recommended that criterion C3c should be retained.

Criterion B defines as “Intra-arterial carotid or vertebral angiography has been performed”. In our study, we found that:

1. In our study, the incidence of headache during the procedure was no statistical difference between cerebral angiography group and coronary intervention group. The incidence of headache within 24 h or within 2–14 days after the procedure was also no statistical difference among the three groups (Table 2). In terms of the characteristics of intraoperative headache, there was no statistical significance in headache onset time, duration and VAS between cerebral angiography group and coronary intervention group (Table 5). The headache onset time, duration and VAS among the three groups were no statistical difference within 24 h or within 2–14 days after the procedure (Table 7 and Table 9). In addition, throbbing pain was more common in all groups no matter intraoperative or postoperative headache.
2. There were both 41.7% (10/24) patients of cerebral angiography group and coronary intervention group, and 25.0% (3/12) patients of extremities arterial intervention group had more than one headache occurred during the 2 weeks’ follow up.
3. Analysis of potential risk factors that may lead to headache showed that previous headache history was a common risk factor among the three groups, and female was a risk factor in cerebral angiography group. By statistical analysis, age, BMI, education, history of allergy, history of intervention, history of tobacco and alcohol, hypertension, cerebral infarction, diabetes were not potential risk factors for headache occurrence among the three groups.

Based on the above analysis, we believe that cerebral angiography headache, coronary intervention-associated headache and extremities arterial intervention -associated headache have similar headache characteristics, suggesting that the occurrence of this kind of headache is not limited to whether intra-arterial carotid or vertebral angiography is performed, coronary intervention and extremities arterial intervention can also lead to similar headache. Criterion B seems to be expanded from “intra-arterial carotid or vertebral angiography has been performed” to “intra-arterial carotid or vertebral angiography or coronary intervention or extremities arterial intervention has been performed”. Considering that *6.7.2 angiography headache* in ICHD-3 belongs to classification 6, namely “*Headache attributed to cranial and/or cervical vascular disorder*”, coronary intervention and extremities arterial intervention do not belong to the category of *Cranial and/or cervical vascular disorder*. Therefore, we recommend that ICHD add categories of “headache due to coronary intervention” and “headache due to extremities arterial intervention”.

The note of *6.7.2 angiography headache* in ICHD-3 states that “there are three recognized (but not separately coded) subforms of 6.7.2 Angiography headache”.

- occurring during angiography, and closely related to contrast injection. This note is an interpretation of criterion C3a and it has been discussed in criterion C3a which is recommended to be retained.
- occurring later, but within 24 h (both these subforms are more common in patients with a history of primary headache, but are distinctly different in character from the primary headache). This note is an interpretation of criterion C1 and it is discussed in criterion C1. We propose to change the definition of “occurring later, but within 24 hours” to “occurring later, but within 1 week after angiography”. Patients with history of headache are more likely to have angiography headache has been established in previous and our studies [2–6, 20]. In our study, the history of headache was a risk factor for angiography headache. As shown in Table 3, the characteristics of the angiography headache can be different from that of the primary headache. Aktan also reported [4] about half of the patients’ angiography headache features were different from their primary headaches. It is recommended to retention “both these subforms are more common in patients with a history of primary headache, but are distinctly different in character from the primary headache”.
- a migraine attack, occurring in a person who has *1. Migraine* and triggered by angiography (in these cases, the patient should have both diagnoses: the appropriate type or subtype of *1. Migraine* and *6.7.2 Angiography headache*).

This note is discussed in criterion C3c and we recommend keeping this note.

Previous studies showed [2, 4] cerebral angiography headache is mostly mild to moderate, throbbing or pressing pain. In our study, cerebral angiography headache was mostly mild-moderate, throbbing or pressing pain, and some patients were accompanied by photophobia, photophobia and visual aura, accompanied by nausea and no vomiting. It is suggested to add a description of the characteristics of headache to the diagnostic criteria, such as: mild-moderate, throbbing or pressing pain with photophobia, nausea and no vomiting. This will increase the clinician’s ability to identify this kind of headache.

Aktan reported 31.0% (13/42) of patients had more than one headache attack after cerebral angiography, and Gil-Gouveia reported that 48.8% (40/82) of patients had recurrent headache [1, 4], The same phenomenon was found in our study, with 41.7% (10/24) patients of cerebral angiography group having two or more headaches. So, we suggested to add to the diagnostic criteria: about one-third of patients may have more than one headache attack.

Based on the above discussion, we suggest that the new diagnostic criteria for *6.7.2 angiography headache* in Table 14.

**Table 14:** Recommended diagnostic criteria for angiography headache

Section 6.7.2: Angiography headache	
Description: Headache caused directly by cerebral angiography.	
Diagnostic criteria:	
A. Any new headache fulfilling criterion C	
B. Intra-arterial carotid or vertebral angiography has been performed	
C. Evidence of causation demonstrated by at least two of the following:	
1. headache has developed during the angiography	
2. headache has developed after the angiography and within 7 days	
3. headache has two of the following sets of characteristics^1^:	
a) developing during contrast injection and lasting <1 hour	
b) developing within 7 days after angiography and a single headache lasting < 24 hours	
c) with pain mostly associated with mild-moderate, throbbing or pressing pain with photophobia, nausea and no vomiting	
D. Not better accounted for by another ICHD-3 diagnosis.	

1There are three recognized (but not separately coded) subforms of 6.7.2 Angiography headache

a) occurring during angiography, and closely related to contrast injection

b) occurring later, but within 7days (both these subforms are more common in patients with a history of primary headache, but are distinctly different in character from the primary headache and about one-third of patients may have more than one headache attack)

c) a migraine attack, occurring in a person who has 1. Migraine and triggered by angiography (in these cases, the patient should have both diagnoses: the appropriate type or subtype of 1. Migraine and 6.7.2 Angiography headache)

## Conclusion

Angiography headache is common in clinical practice but has received little attention. In our study, the incidence of headache was 22.4% in cerebral angiography group, 23.8% in coronary intervention group, and 16.9% in extremities arterial intervention group. There was no statistical significance in the incidence of headache among the three groups. Female and history of headache were risk factors in cerebral angiography group, and history of headache was a risk factor in coronary intervention group and extremities arterial intervention group. There were no differences in headache onset time, duration and VAS of intraoperative headache between cerebral angiography group and coronary intervention group. The headache onset time, duration and VAS of postoperative headache were similar in three groups. By discussing the diagnosis criteria of *6.7.2 angiography headache* in ICHD-3, we believe that there are problems in the applicability of the diagnostic criteria and it is easy to miss diagnosis, so it should be revised according to the current literature and research. In addition, it is suggested to add two new definitions and diagnostic criteria of “headache due to coronary intervention” and “headache due to extremities arterial intervention” in ICHD-3.

## Data Availability

The datasets used and analyzed during the present study are available from the corresponding authors on reasonable request.

## Abbreviations

AH: Angiography headache
ICHD-3: International Classification of Headache Disorders, 3rd edition
DSA: Digital subtraction angiography
PLA: People’s Liberation Army
CAG: Coronary angiography
PCI: Percutaneous coronary intervention
BMI: Body mass index
VAS: Visual analog scale
SD: Standard deviation
HA: Headache
AG: Angiography
M: Migraine
TTH: Tension-type headache
N/A: Not applicable
IQR: Interquartile range
AMI: Acute myocardial infarction
EAI: Extremities arterial intervention
OR: Odds ratio
CI: Confidence interval
